# Mathematical Modeling of IOL Power Changes After Pupil Dilation Based on Multifactorial Analysis

**DOI:** 10.1155/joph/8897808

**Published:** 2026-06-25

**Authors:** Shasha Xue, Xinyu Wang, Yunxiao Wang, Fenglei Wang, Licun Wang, Xiaomei Wan, Ling Wang

**Affiliations:** ^1^ Department of Ophthalmology, The Affiliated Hospital of Qingdao University, Qingdao, China, qdu.edu.cn

**Keywords:** IOL power, mathematical modeling, multifactorial analysis, ocular parameters, pupil dilation

## Abstract

**Purpose:**

This study aimed to explore the factors affecting the changes in intraocular lens (IOL) power after pupil dilation in patients with age‐related cataract and to construct linear regression models.

**Methods:**

A total of 167 patients with age of 66.47 ± 8.63 (years) were included in this study to evaluate whether pupil dilation led to significant changes in axial length (AL), corneal curvature (K), central anterior chamber depth (ACD), central corneal thickness (CCT), lens thickness (LT), white‐to‐white corneal diameter (WTW), relative lens position (RLP), and the values of IOL power predicted using different formulas. Pearson linear correlation analysis was performed between dilation‐induced IOL power changes (ΔIOL) and other changes in ocular parameters (ΔAL, ΔK, ΔACD, ΔCCT, ΔLT, ΔWTW, and ΔRLP), after which regression models were established using ΔIOL and its correlates.

**Results:**

For each formula, a prediction model of ΔIOL was established using the regression coefficients: ΔIOL_Haigis_ = 0.06 − 3.381 ΔAL − 1.335 ΔK; ΔIOL_Hoffer-Q_ = −3.547 ΔAL − 1.318 Δ*K*; ΔIOL_SRK-T_ = −3.105 ΔAL − 1.019 Δ*K*; ΔIOL_Holladay-1_ = −3.697 ΔAL − 1.276 Δ*K*; ΔIOL_Holladay-2_ = 0.099 − 3.484 ΔAL − 1.235 Δ*K* − 0.279 ΔWTW; ΔIOL_Barrett-II_ = 0.071 − 3.702 ΔAL − 1.147 Δ*K* + 0.069 ΔWTW.

**Conclusion:**

Multivariate mathematical models of ΔIOL suggest that IOL power changes are mainly related to the changes in AL and *K* and that the values of ΔAL, Δ*K*, and ΔWTW can be used to predict ΔIOL that is calculated by various formulas.

**Trail Registration:** Chinese Registry of Clinical Trials: ChiCTR2200056856

## 1. Introduction

Cataract surgery is undergoing a significant transition from merely restoring vision to focusing on refractive outcomes. The accurate calculation of intraocular lens (IOL) power is crucial for ensuring the clinical effect of cataract surgery. However, there are still certain challenges in the precise calculation of IOL power in clinical practice. Previous studies have shown that biometric errors, such as those in anterior chamber depth (ACD), axial length (AL), and corneal curvature (*K*), may lead to errors in IOL power calculation by 42%, 36%, and 22%, respectively [1]. In addition, the proportion of postoperative refractive error (PE) within ± 0.5 D after cataract surgery could be 73.7% [2], indicating that there is still room for improvement in the accuracy of IOL power calculation.

Of course, careful preoperative examination of the fundus helps to predict postoperative vision after cataract surgery. However, whether other preoperative eye tests, such as measuring IOL power and examining corneal morphology, can be conducted after pupil dilation is a concern. Studies have shown that pupil dilation affects the change of ocular parameters and IOL power in patients [3–5]; calculations based on various IOL power formulas showed that 23.6%–40.7% of participants experienced IOL power changes greater than 0.50 diopters (D) after pupil dilation, and 0.7%–1.4% experienced changes greater than 1.0 D [6].

AL and corneal curvature (*K*) are essential parameters in various IOL power calculation formulas. The third‐generation formulas (Holladay‐1, SRK‐T, Hoffer‐Q), which are widely used today, require only AL and *K* values to calculate IOL power. The fourth‐generation Haigis formula utilizes AL, *K*, and central ACD to compute IOL power. The Holladay‐2 formula incorporates AL, *K*, ACD, lens thickness (LT), white‐to‐white corneal diameter (WTW), and age, while the fifth‐generation Barrett‐II formula uses parameters such as AL, *K*, ACD, LT, and WTW, theoretically leading to increasingly accurate IOL power calculations [7, 8]. However, there are no reports on whether the dilation‐induced IOL power changes calculated by different formulas are consistent, whether these changes correlate with the mentioned parameters to consistent degrees among these formulas, or whether it is possible to identify sensitive parameters that significantly impact each formula and then to construct linear regression models for predicting IOL dilation‐induced power changes. In this study, we analyzed and compared changes in IOL power and related ocular parameters after pupil dilation, assessed the correlation between IOL power changes calculated by different formulas and parameter changes, and constructed mathematical models to provide more precise target refraction for selecting IOLs for cataract patients.

## 2. Methods

### 2.1. Subjects

A total of 167 patients with age‐related cataracts who were scheduled to undergo right‐eye cataract surgery by ultrasonic emulsification combined with IOL implantation in the Affiliated Hospital of Qingdao University from June 2022 to June 2023 were included in this study. All the patients had a clear diagnosis before the operation and were aged ≥ 45 years, with preoperative intraocular pressure of 10–21 mmHg, nondilated pupil diameter (PD) ≤ 4.0 mm, and pupillary light reflex. The exclusion criteria were as follows: (1) patients with other comorbid ocular diseases, such as corneal disorders, lens dislocation, active ocular inflammation, history of previous ocular trauma, or intraocular surgery and patients with previous refractive surgery; (2) patients with conditions such as anterior and posterior iris synechiae that affect pupil mobility; (3) patients with poor visual fixation and poor cooperation. This study was approved by the Ethics Committee of the Affiliated Hospital of Qingdao University on February 21, 2022. This study adhered to the Declaration of Helsinki, and all participants signed an informed consent form.

### 2.2. Data Analysis

All patients were routinely examined preoperatively for best‐corrected visual acuity (Topcon Ltd, CV‐5000, Japan), intraocular pressure (Goldmann applanation tonometer), slit lamp examination (Haag‐Streit Ltd, BM 900, Switzerland), and fundus examination (Volk Ltd, VSFNC, USA). IOL Master measurements (IOLMaster 700, Carl Zeiss Medifec AG, Germany) were performed by the same experienced operator before and after pupil dilation under the same lighting conditions, with the operator measuring three consecutive times and the system automatically calculating the average, thereby obtaining the values of PD, AL, *K*, ACD, central corneal thickness (CCT), LT, WTW, and IOL power calculated using different formulas. Setting the target refractive error to 0 D and choosing TECNIS 1 ZCB00 (AMO) as the target IOL model for each eye, we calculated IOL power using the third‐generation IOL formulas Holladay‐1, SRK‐T, Hoffer‐Q, the fourth‐generation IOL formulas Holladay‐2, Haigis, and the fifth‐generation IOL formula Barrett‐II. Pupils were dilated by instilling tropicamide eye drops (0.5%, Zhuo Bi An, Shenyang Xingqi Eye Medicine, China) into the conjunctival sac thrice at five‐minute intervals, with eyes closed for 40 min after the last application until the PD was ≥ 6 mm, followed by another IOLMaster examination. The examination room was completely shaded to avoid natural light interference, and redundant indoor lights were turned off. A calibrated lux meter was used to quantitatively monitor the ambient illuminance at the subject’s eye level, and the lighting intensity was maintained stably at 30–50 lux throughout the test. The lighting layout, background brightness, and environmental reflection remained consistent during the two detection periods, enabling unified lighting evaluation and standardized operation.

### 2.3. Statistical Methods

The SPSS22.0 software was used for statistical analysis, the GraphPad Prism software was used to draw statistical graphs, and the measurement results were expressed as the mean ± standard deviation (*x* ± *s*). The Kolmogorov–Smirnov test was used to check whether the data were normally distributed. A paired *t*‐test was performed for pre‐ and postdilation data if they obeyed normal distribution; otherwise, the Wilcoxon signed rank‐sum test was performed, with differences deemed significant at *p* < 0.05.

Sample size estimation was performed in accordance with the principles of sample size calculation for multiple linear regression. Combined with the self‐controlled before‐and‐after study design of this research, the minimum sample size was estimated based on the standard of 10 samples per independent variable. Considering a 15% sample loss rate, the final sample size requirement was determined. A total of 167 subjects were actually included in the study, which was much higher than the minimum sample size requirement, ensuring that the statistical power was ≥ 0.8. Pearson’s correlation analysis was performed between dilation‐induced ΔIOL and other ocular parameter changes to establish linear regression models.

## 3. Results

### 3.1. Comparison of Ocular Parameters and IOL Powers Before and After Pupil Dilation

A total of 167 eyes were included in this study, with a male/female ratio of 79/88, patient age of 66.47 ± 8.63 (years), preoperative best‐corrected visual acuity of 0.35 ± 0.12, and intraocular pressure of 15.33 ± 1.94 (mmHg).

**FIGURE 1:**
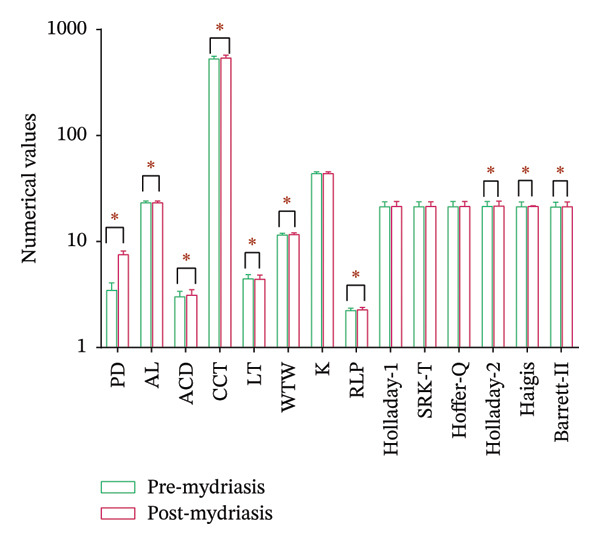
Ocular parameters and IOL powers with different formulas before and after mydriasis; *p* < 0.05.

The PD, ACD, CCT, WTW, and RLP increased and AL and LT decreased during the period after pupil dilation compared with the period before pupil dilation, and the differences were significant (All *p* ≤ 0.001); the *K* values before and after pupil dilation were (44.15 ± 1.44) D and (44.13 ± 1.43) D, respectively, and the differences were not significant, but *K* tended to decrease after pupil dilation. The IOL power calculated using different formulas increased during the period after pupil dilation compared with the period before pupil dilation, but the increase was not significant in the case of the third‐generation formulas Holladay‐1, SRK‐T, and Hoffer‐Q (*p* = 0.056, 0.052, and 0.056, respectively); in contrast, the increase was significant in the case of the fourth‐generation formulas Holladay‐2 and Haigis, as well as the fifth‐generation formula Barrett‐II (All *p* ≤ 0.001) (Table 1, Figure 1).

**TABLE 1:** Comparison of ocular parameters and IOL powers before and after pupil dilation (*x* ± *s*).

	Predilation	Postdilation	*p*
PD (mm)	3.48 ± 0.60	7.55 ± 0.59	≤ 0.001
AL (mm)	23.37 ± 0.88	23.36 ± 0.88	≤ 0.001
ACD (mm)	3.02 ± 0.38	3.14 ± 0.38	≤ 0.001
CCT (μm)	532.43 ± 29.57	542.18 ± 31.05	≤ 0.001
LT (mm)	4.47 ± 0.40	4.44 ± 0.40	≤ 0.001
WTW (mm)	11.60 ± 0.40	11.70 ± 0.42	≤ 0.001
*K* (D)	44.15 ± 1.44	44.13 ± 1.43	0.384
RLP	2.25 ± 0.10	2.29 ± 0.10	≤ 0.001
Holladay‐1 (D)	21.48 ± 2.32	21.51 ± 2.35	0.056
SRK‐T (D)	21.48 ± 2.24	21.53 ± 2.26	0.052
Hoffer‐Q (D)	21.48 ± 2.43	21.53 ± 2.45	0.056
Holladay‐2 (D)	21.62 ± 2.33	21.73 ± 2.35	≤ 0.001
Haigis (D)	21.46 ± 2.24	21.56 ± 0.27	≤ 0.001
Barrett‐II (D)	21.36 ± 2.20	21.48 ± 2.23	≤ 0.001

### 3.2. Correlation Analysis of Dilation‐Induced IOL Power Changes (ΔIOL) With Respect to Various Ocular Parameters

Dilation‐induced ΔIOL, as calculated using different formulas, i.e., ΔIOL_Holladay-1_, ΔIOL_SRK-T_, ΔIOL_Hoffer-Q_, ΔIOL_Holladay-2_, ΔIOL_Haigis_, and ΔIOL_Barrett-II_, correlated negatively with dilation‐induced changes in AL (ΔAL) (*r* = −0.344, *p* ≤ 0.001; *r* = −0.413, *p* ≤ 0.001; *r* = −0.383, *p* ≤ 0.001; *r* = −0.403, *p* ≤ 0.001; *r* = −0.367, *p* ≤ 0.001; *r* = −0.412, *p* ≤ 0.001; respectively).

 ΔIOL_Holladay-1_, ΔIOL_SRK-T_, ΔIOL_Hoffer-Q_, ΔIOL_Holladay-2_, ΔIOL_Haigis_, and ΔIOL_Barrett-II_ exhibited a significant negative correlation with dilation‐induced changes in *K*‐values (Δ*K*) (*r* = −0.799, *p* ≤ 0.001; *r* = −0.926, *p* ≤ 0.001; *r* = −0.936, *p* ≤ 0.001; *r* = −0.882, *p* ≤ 0.001; *r* = −0.935, *p* ≤ 0.001; *r* = −0.904, *p* ≤ 0.001, respectively).
 ΔIOL_Holladay-1_, ΔIOL_SRK-T_, ΔIOL_Hoffer-Q_, ΔIOL_Holladay-2_, ΔIOL_Haigis_, and ΔIOL_Barrett-II_ correlated negatively with dilation‐induced changes in CCT (ΔCCT) (*r* = −0.220, *p* = 0.004; *r* = −0.248, *p* = 0.001; *r* = −0.250, *p* = 0.001; *r* = −0.232, *p* = 0.003; *r* = −0.253, *p* = 0.001; *r* = −0.246, *p* = 0.001; respectively).
 ΔIOL_Holladay-2_ and ΔIOL_Barrett-II_ correlated with dilation‐induced changes in WTW (ΔWTW), with the correlation being negative for ΔIOL_Holladay-2_ (*r* = −0.280, *p* ≤ 0.001) and positive for ΔIOL_Barrett-II_ (*r* = 0.231, *p* ≤ 0.001); ΔIOL_Holladay-1_, ΔIOL_SRK-T_, ΔIOL_Hoffer-Q_, and ΔIOL_Haigis_ had no significant correlation with ΔWTW (|*r*| < 0.05, *p* > 0.05).

**FIGURE 2:**
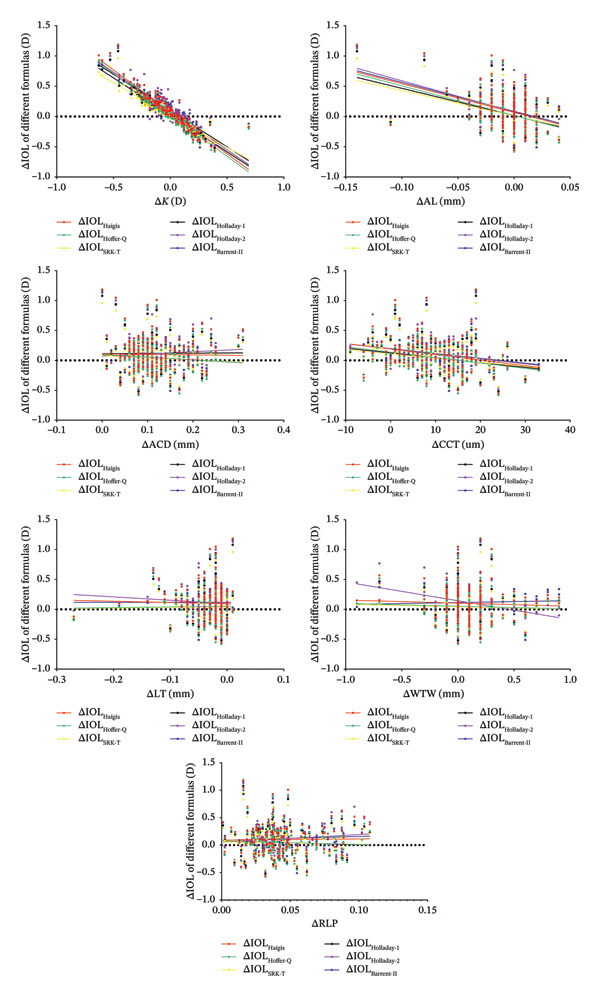
Scatter plot of correlation between changes in ocular parameters and IOL power before and after mydriasis.

None of ΔIOL_Holladay-1_, ΔIOL_SRK-T_, ΔIOL_Hoffer-Q_, ΔIOL_Holladay-2_, ΔIOL_Haigis_, and ΔIOL_Barrett-II_ correlated with dilation‐induced changes in ACD (ΔACD), changes in LT (ΔLT), and changes in RLP (ΔRLP) (All |*r*| < 0.20, *p* > 0.05) (Table 2, Figure 2).

**TABLE 2:** Correlation analysis between ΔIOL with different formulas and the changes in relevant ocular parameters.

		ΔHolladay‐1	ΔSRK‐T	ΔHoffer‐Q	ΔHolladay‐2	ΔHaigis	ΔBarrett‐II
ΔAL	Pearson correlation	−0.344^∗^	−0.413^∗^	−0.383^∗^	−0.403^∗^	−0.367^∗^	−0.412^∗^
	Sig. (double tailed)	0.000	0.000	0.000	0.000	0.000	0.000

ΔK	Pearson correlation	−0.799^∗^	−0.926^∗^	−0.936^∗^	−0.882^∗^	−0.935^∗^	−0.904^∗^
	Sig. (double tailed)	0.000	0.000	0.000	0.000	0.000	0.000

ΔACD	Pearson correlation	−0.014	−0.092	−0.094	0.077	−0.017	0.009
	Sig. (double tailed)	0.853	0.236	0.228	0.326	0.826	0.907

ΔLT	Pearson correlation	−0.015	0.018	0.015	−0.072	−0.026	0.000
	Sig. (double tailed)	0.852	0.819	0.850	0.355	0.735	0.998

ΔWTW	Pearson correlation	−0.041	−0.046	−0.040	−0.280^∗^	−0.045	0.231^∗^
	Sig. (double tailed)	0.598	0.554	0.607	0.000	0.567	0.000

ΔCCT	Pearson correlation	−0.220^∗^	−0.248^∗^	−0.250^∗^	−0.232^∗^	−0.253^∗^	−0.246^∗^
	Sig. (double tailed)	0.004	0.001	0.001	0.003	0.001	0.001

ΔRLP	Pearson correlation	0.020	−0.047	−0.052	0.111	0.018	0.061
	Sig. (double tailed)	0.795	0.550	0.503	0.154	0.820	0.431

^∗^Significant at the 0.01 level (double‐tailed).

### 3.3. Mathematical Models of ΔIOL With Respect to Ocular Parameters of Significant Correlation

Correlation analysis of dilation‐induced ΔIOL (calculated using various formulas) with respect to each ocular parameter revealed that ΔIOL_Holladay-1_ correlated with Δ*K*, ΔAL, and ΔCCT. A linear regression model was established based on these relationships, indicating that the correlation between ΔIOL_Holladay-1_ and ΔCCT was spurious (*p* = 0.779). A prediction model of ΔIOL_Holladay-1_ was established using the regression coefficients: ΔIOL_Holladay-1_ = −3.697 ΔAL − 1.276 Δ*K*, this model explained 70.1% of the variation in dilation‐induced ΔIOL_Holladay-1_ (*R* ^2^ = 0.701).

Correlation analysis also revealed that ΔIOL_SRK-T_ correlated with Δ*K*, ΔAL, and ΔCCT. A linear regression model was established based on the three relationships which indicated that the correlation between ΔIOL_SRK-T_ and ΔCCT was spurious (*p* = 0.715). A prediction model of ΔIOL_SRK-T_ was established using the regression coefficients: ΔIOL_SRK-T_ = −3.105 ΔAL − 1.019 Δ*K*, the model explained 95.2% of the variation in dilation‐induced ΔIOL_SRK-T_ (*R* ^2^ = 0.952).

Moreover, correlation analysis revealed that ΔIOL_Hoffer-Q_ correlated with Δ*K*, ΔAL, and ΔCCT. A linear regression model was established based on the three relationships which indicated that the correlation between ΔIOL_Hoffer-Q_ and ΔCCT was spurious (*p* = 0.719). A prediction model of ΔIOL_Hoffer-Q_ was established using the regression coefficients: ΔIOL_Hoffer-Q_ = −3.547 ΔAL − 1.318 Δ*K*, the model explained 95.2% of the variation in dilation‐induced ΔIOL_Hoffer-Q_ (*R* ^2^ = 0.952).

 ΔIOL_Holladay-2_ was correlated with ΔK, ΔAL, ΔCCT, and ΔWTW. A linear regression model was established based on the four relationships which indicated that the correlation between ΔIOL_Holladay-2_ and ΔCCT was spurious (*p* = 0.929). A prediction model of ΔIOL_Holladay-2_ was established using the regression coefficients: ΔIOL_Holladay-2_ = 0.099 − 3.484 ΔAL − 1.235 Δ*K* − 0.279 ΔWTW, the model explained 92.9% of the variation in dilation‐induced ΔIOL_Holladay-2_ (*R* ^2^ = 0.929).
 ΔIOL_Haigis_ correlated with Δ*K*, ΔAL, and ΔCCT. A linear regression model was established based on the three relationships which indicated that the correlation between ΔIOL_Haigis_ and ΔCCT was spurious (*p* = 0.594). A prediction model of ΔIOL_Haigis_ was established using the regression coefficients: ΔIOL_Haigis_ = 0.06 − 3.381 ΔAL − 1.335 Δ*K*, the model explained 94.2% of the variation in dilation‐induced ΔIOL_Haigis_ (*R* ^2^ = 0.942).
 ΔIOL_Barrett-II_ correlated with ΔK, ΔAL, ΔCCT, and ΔWTW. A linear regression model was established based on the four relationships which indicated that the correlation between ΔIOL_Barrett-II_ and ΔCCT was spurious (*p* = 0.667). A prediction model of ΔIOL_Barrett-II_ was established using the regression coefficients: ΔIOL_Barrett-II_ = 0.071 − 3.702 ΔAL − 1.147 Δ*K* + 0.069 ΔWTW, the model explained 91.6% of the variation in dilation‐induced ΔIOL_Barrett-II_ (*R* ^2^ = 0.916) (Table 3).

**TABLE 3:** Linear regression modeling.

Regression model	*R* ^2^	Independent variable	Coefficient	*t*	Sig. (*p*)
1^a^	0.701	Constant	−0.011	−0.492	0.623
		ΔAL	−3.697	−5.877	≤ 0.001
		Δ*K*	−1.276	−17.218	≤ 0.001
		ΔCCT	−0.001	−0.281	0.779

2^b^	0.952	Constant	0.005	0.833	0.406
		ΔAL	−3.105	−17.922	≤ 0.001
		Δ*K*	−1.019	−49.933	≤ 0.001
		ΔCCT	0.000	−0.366	0.715

3^c^	0.952	Constant	0.007	0.834	0.405
		ΔAL	−3.547	−16.058	≤ 0.001
		Δ*K*	−1.318	−50.645	≤ 0.001
		ΔCCT	0.000	−0.361	0.719

4^d^	0.929	Constant	0.099	9.736	≤ 0.001
		ΔAL	−3.484	−12.896	≤ 0.001
		Δ*K*	−1.235	−39.109	≤ 0.001
		ΔCCT	0.000	−0.089	0.929
		ΔWTW	−0.279	−11.800	≤ 0.001

5^e^	0.942	Constant	0.060	6.607	≤ 0.001
		ΔAL	−3.381	−13.670	≤ 0.001
		Δ*K*	−1.335	−45.831	≤ 0.001
		ΔCCT	0.000	−0.534	0.594

6^f^	0.916	Constant	0.071	7.040	≤ 0.001
		ΔAL	−3.702	−13.791	≤ 0.001
		Δ*K*	−1.147	−36.545	≤ 0.001
		ΔCCT	0.000	−0.431	0.667
		ΔWTW	0.069	2.955	0.004

*Note:* Dependent variable.

^a^ΔIOL_Holladay-1_ (ΔIOL_Holladay-1_ = −3.697 ∗ ΔAL − 1.276 ∗ Δ*K*).

^b^ΔIOL_SRK-T_ (ΔIOL_SRK-T_ = −3.105 ∗ ΔAL − 1.019 ∗ Δ*K*).

^c^ΔIOL_Hoffer-Q_ (ΔIOL_Hoffer-Q_ = −3.547 ∗ ΔAL − 1.318 ∗ Δ*K*).

^d^ΔIOL_Holladay-2_ (ΔIOL_Holladay-2_ = 0.099 − 3.484 ∗ ΔAL − 1.235 ∗ Δ*K* − 0.279 ∗ ΔWTW).

^e^ΔIOL_Haigis_ (ΔIOL_Haigis_ = 0.06 − 3.381 ∗ ΔAL − 1.335 ∗ Δ*K*).

^f^ΔIOL_Barrett-II_ (ΔIOL_Barrett-II_ = 0.071 − 3.702 ∗ ΔAL − 1.147 ∗ Δ*K* + 0.069 ∗ ΔWTW).

## 4. Discussion

The pursuit of precise postoperative refractive outcomes by modern cataract patients compels ophthalmologists to focus on the accuracy of postoperative refraction. In this regard, preoperative pupil dilation is essential for a detailed examination of the patient’s eye condition. Pupil dilatation may affect certain ocular biologic parameters, which in turn affects the accuracy of IOL power calculated using various IOL formulas [9–11]. With the advent and application of ocular biometric devices with increasingly high measurement accuracy, factors affecting the postoperative refractive outcome, including AL, corneal curvature, ACD, LT, WTW corneal diameter, and age, are being increasingly emphasized. IOL formulas have evolved from the first to the fifth generation, significantly enhancing overall visual outcomes after cataract surgery [12, 13].

Currently, numerous studies [9–14] are exploring the impact of pupil dilation on ocular parameters and the accuracy of IOL power calculated using various formulas. Clinically, IOL power is adjusted in increments or decrements of 0.5 D; therefore, scholars generally take the change of 0.5 D or 1.0 D as the baseline to discuss the proportion of IOL power changes caused by pupil dilation and their clinical significance, though this approach may not be very rigorous in actual clinical practice due to individual differences. Pupil dilation has no statistically significant effect on IOL power calculated using third‐generation formula IOLs but does affect calculations based on fourth‐ and fifth‐generation formulas [15], which is consistent with the results of this study. A difference of 0.1 D in IOL power is equivalent to approximately 0.067 D in spectacle power, and this difference can significantly affect the refractive outcome of cataract surgery [16]. Building on the known effects of pupil dilation on IOL power, this study further investigated which sensitive indicators may be influenced by dilation and constructed mathematical models to predict changes in IOL power across different formulas based on these indicators. The changes in calculated IOL power can provide clues as to whether patients need to undergo follow‐up examinations after their pupils return to normal, thereby providing cataract patients with more precise target refraction and improved visual quality.

In this study, the ACD increased significantly after pupil dilation compared to before pupil dilation. This increase was partly due to the tightening of the suspensory ligaments following ciliary muscle paralysis, leading to the lens becoming thinner and flatter. Additionally, pupil dilation reduced the contact area between the iris and the lens, decreased the resistance to aqueous humor flow from the posterior to the anterior chamber, and caused the iris to flatten and shift posteriorly. Simultaneously, the relative position of the lens increased, further indicating that the backward movement of the lens‐iris diaphragm after pupil dilation led to the posterior shift of the anterior segment and an increase in the central ACD. The CCT increased after pupil dilation. Either dilation or constriction of the pupil may cause an increase in the CCT; however, the mechanism remains unclear. This change is thought to be possibly related to temporary alterations in the corneal epithelium and stroma caused by the preservatives in the eye drops. Additionally, keeping the eyes closed for a prolonged period after administering the drops may further influence corneal epithelial metabolism [17–19].

In this study, the corneal *K*‐value was (44.15 ± 1.44) D before ciliary muscle paralysis and (44.13 ± 1.43) D after ciliary muscle paralysis; i.e., there was a tendency for the corneal *K*‐value to decrease after pupil dilation. Tuncer et al.’s study observed that the mean corneal *K*‐value of the age group of 50–60 years significantly decreased by 0.054 D after pupil dilation [20]. Peñaranda reported that after ciliary muscle paralysis, corneal power decreased by 0.15 D [21]. Meanwhile, Cheng and Hsieh suggested that ciliary muscle paralysis caused a decrease of 0.032 D in the corneal *K*‐value [22]. This phenomenon may be related to the decrease in the centripetal force of the scleral spur due to ciliary muscle paralysis, which causes the flattening of the cornea [12, 23–25]. However, the magnitudes of observed corneal *K*‐value changes differ among different studies, which may be related to the relatively limited reliability of the IOL Master in measuring corneal morphology. It is recommended to utilize a corneal topography system to further verify these findings.

Khambhiphant et al. observed that pupil dilation had no significant effect on AL [13, 14, 26–28]. However, Xi et al. concluded that in elderly patients, pupil dilation causes an increase in AL, and they conjectured that the ciliary body moves backward after ciliary muscle paralysis, creating a force that pushes toward the vitreous cavity, resulting in temporary AL elongation, while an increase in the CCT may also cause an AL increase [7, 20]. However, this study observed a decrease in AL in the period after pupil dilation compared with the period before pupil dilation, which may be due to the flattening of the cornea after ciliary muscle paralysis, thereby leading to a reduction in AL. A 0.01 mm difference in AL can cause a change in IOL power of about ± 0.028 D [6]. Therefore, even if the amount of change in AL after pupil dilation is small, it should be taken seriously by clinicians.

Tuncer concluded that pupil dilation significantly increased WTW in the age groups of 10–20 years and 30–40 years, whereas there was no significant difference in WTW for the age group of 50–60 years [20]. In this study, an increase in WTW after dilation was observed, consistent with the findings of Xi, Chang, Arici, and Arriola‐Villalobos, among others [7, 25, 29, 30]. The mechanism behind these results is unclear. Some scholars suggest that optical biometric devices measure WTW using an image analysis system. These devices rely on the sudden contrast change from the lighter sclera to the darker cornea to digitally locate the corneal edges. After pupil dilation, the contrast between the dark iris and the lighter sclera becomes more pronounced, whereas their boundaries are not distinct, with contrast differences varying with lighting and image quality. In such cases, the WTW measurements after pupil dilation may be larger than previous measurements [28, 31, 32].

Although there was no significant difference in IOL power calculated using third‐generation formulas (Holladay‐1, SRK‐T, Hoffer‐Q) between the period before and the period after pupil dilation, significant differences were observed for fourth‐generation formulas (Haigis, Holladay‐2) and fifth‐generation formulas (Barrett‐II). Moreover, there was a tendency for calculated IOL power to increase after pupil dilation compared to the predilation values, consistent with the finding by Rodriguez‐Raton [33]. The changes in IOL power after pupil dilation, calculated using various formulas, relative to the predilation values were taken as a separate dataset to investigate which of the ocular parameters affected by dilation had the most profound impact on IOL power. The results showed that for the Holladay‐1, SRK‐T, Hoffer‐Q, and Haigis formulas, ΔAL and Δ*K* were the main factors affecting ΔIOL, both negatively correlating with ΔIOL; for the Holladay‐2 and Barrett‐II formulas, ΔAL and Δ*K* were the main factors affecting ΔIOL, both negatively correlating with ΔIOL, while ΔWTW was a secondary factor, which negatively correlated with the ΔIOL derived from the Holladay‐2 formulas and positively correlated with ΔIOL in the case of the Barrett‐II formula. For each IOL formula, a mathematical model of ΔIOL with respect to its correlates was established. This model showed that the coefficients of ΔAL ranged from −3 to −4, and the coefficients of Δ*K* ranged from −1 to −2. AL, which encompasses ACD, LT, and vitreous cavity depth, can change IOL power by a factor of 2.5–3, and the change in corneal power can change IOL power at a nearly 1:1 ratio [34, 35], which is consistent with the results of the mathematical models established in this study.

This study was the first of its kind to examine the changes in IOL power calculated using various formulas between the period before and the period after pupil dilation, identify the sensitive ocular parameters with changes that correlate with the IOL power changes, and then construct mathematical models of IOL power changes with the correlates. Through the linear regression model, we successfully quantified the magnitude of this change and the degree of influence of each relevant factor on the change. The results showed that the changes in *K* and AL caused by pupil dilation were the main factors causing the changes in IOL power and that the changes in WTW were the secondary factor causing the changes in IOL power in the case of the Barrett‐II and Holladay‐2 formulas, while the changes in ACD and LT were not significant factors. And after reinserting the changes in AL, *K* values, and WTW caused by pupil dilation into different formula mathematical models, the resulting changes in IOL powers were consistent with the actual measurements obtained from the IOLMaster examination, further indicating that these mathematical models are reliable for estimating the changes in IOL powers caused by dilation. In clinical practice, it is possible to predict the changes in IOL power through the changes in sensitive factors, to give a clue to the accuracy of relevant examinations under different pupil states, and to provide cataract patients with more accurate target refraction and postoperative visual outcomes. However, there are some limitations in this study. The patients included were elderly cataract patients with ALs within the normal range. However, different IOL power calculation formulas exhibit distinct advantages in eyes with varying ALs. The Hoffer‐Q formula demonstrates higher accuracy in short eyes [36], whereas the Barrett‐II and Holladay‐1 formulas are more reliable for IOL calculation in long eyes [37]. These findings have been well validated in previous studies. Future research should include participants with different ALs and from various age groups to further explore the applicability and accuracy of the digital model.

## 5. Conclusions

1. Pupil dilation has minimal effect on the IOL power calculations with third‐generation IOL formulas and a moderate effect for fourth‐ and fifth‐generation formulas. It is recommended that IOL power be redetermined after pupil dilation.
2. Changes in *K*‐value due to pupil dilation and changes in AL are the main factors causing changes in calculated IOL power, while changes in WTW are the secondary factor in the case of the Barrett II and Holladay 2 formulas, with changes in ACD and LT being nonsignificant factors.
3. The mathematical models of dilation‐induced IOL power changes, which were established through multivariate analysis, suggested that IOL power changes are mainly related to the changes in AL and *K*. The values of ΔAL, Δ*K*, and ΔWTW can be used to predict the changes in IOL power calculated by various formulas.

## Funding

This study was supported by the Qingdao Key Health Discipline Development Fund, 10.13039/100019558.

## Ethics Statement

The research adhered to the tenets of the Declaration of Helsinki and was approved by the Affiliated Hospital of Qingdao University Ethics Committee. All patients were adequately informed about the study, and the informed consent was obtained from all of the participants. It is available for review by the Editor of this journal if necessary.

## Conflicts of Interest

The authors declare no conflicts of interest.

## Data Availability

The data analyzed during the current study are available from the corresponding author on reasonable request.
